# Feasibility of implementing a decision aid for advanced cancer patients in a comprehensive cancer centers' outpatient setting: Valuable lessons learned

**DOI:** 10.1002/cam4.70127

**Published:** 2024-12-17

**Authors:** K. Laryionava, R. Boekels, J. Schildmann, M. Wensing, U. Wedding, B. Surmann, K. Mehlis, C. Gebel, M. Cinci, K. Krug, E. C. Winkler

**Affiliations:** ^1^ Department of Medical Oncology, Medical Faculty Heidelberg, Section Translational Medical Ethics, National Center for Tumor Diseases, NCT Heidelberg, a partnership between DKFZ and Heidelberg University Hospital Heidelberg University Heidelberg Germany; ^2^ Institute for History and Ethics of Medicine, Centre for Health Sciences Martin Luther University Halle‐Wittenberg (Saale) Halle (Saale) Germany; ^3^ Department of General Practice and Health Services Research Heidelberg University Hospital Heidelberg Germany; ^4^ Department of Internal Medicine II (Palliative Care) University of Jena Jena Germany; ^5^ Health Economics and Health Care Management Bielefeld University Bielefeld Germany

**Keywords:** advanced cancer, decision aid, forgoing of cancer treatment, implementation research, qualitative study

## Abstract

**Background:**

Decision aids (DAs) have been proposed as tools to empower patients in decision‐making. However, implementing DA for decisions in advanced cancer is challenging. This study focuses on perspectives of oncologists and other healthcare providers on the hindering and facilitating factors for implementing a customizable DA. This DA is designed to support discussions about whether patients with advanced, incurable cancers should pursue or forgo further anticancer treatment.

**Methods:**

This qualitative study utilized content analysis following Sandelowski's approach on two different datasets derived from research projects on decision‐making in advanced cancer. To inform the development of a DA and understand challenges related to its implementation, we conducted semi‐structured interviews with 24 oncologists (Dataset 1) and three focus groups with various healthcare professionals (*n* = 19) and in addition post‐implementation semi‐structured interviews with oncologists who used the DA in conversations with their cancer patients (*n* = 5) (Dataset 2). Furthermore, the insights from process evaluation of developing and implementing the DA in an outpatients' settings was incorporated, enriching the analytical framework.

**Results:**

Two overall themes emerged: (1) Tension between standardizing the use of a DA and responding to individual patient needs. To address this conflict, a two‐part DA was developed, combining structured elements with flexibility. (2) Prerequisites for the use of the DA in outpatients' settings: Senior physicians' support, education in palliative care options and in the use of the DA, and organizational conditions.

**Conclusion:**

Oncologists identified both structural and content‐related aspects for a successful implementation of a DA for patients with advanced cancer. While the content‐related aspects were factored into the development of the DA, structural (organizational) issues need to be especially focused on during implementation.

## 1 INTRODUCTION

Shared decision making (SDM) is a central part of high‐quality care of patients with advanced cancer. SDM is a collaborative process where physicians and patients work together to make decisions about the patient's treatment and care. This approach emphasizes the exchange of information between oncologists and patients, allowing patients to actively participate in their treatment choices. SDM prioritizes the patient's individual preferences, expectations regarding treatment goals, values, and circumstances to arrive at a mutually agreed‐upon treatment plan.^1^, ^2^

The significance of SDM becomes even more pronounced in the context of advanced cancer, when the typical guideline based treatment options have been exhausted.

In this phase, patients often face difficult choices about whether to continue or forgo anticancer treatment that means focusing on the best supportive care or opting for more experimental treatment options, such as participating in clinical trials or pursuing off‐label use supported by a molecular rationale.^3^ Patients are frequently faced with the challenge to balance marginal effects on gaining life time, side effects, and quality of life considerations.^2^

To enhance SDM, empirical research suggests that decision aids (DAs) can serve as valuable tools in empowering cancer patients in decision‐making about forgoing anticancer treatment in far advanced cancer.^4^

The use of DAs has been shown to improve cancer patients' understanding of prognosis, satisfaction with communication, compliance, confidence in decisions, and in some studies, reduce decisional conflicts.^5^, ^6^

Despite the undeniable benefits of DAs, several challenges and barriers were shown to hinder their successful implementation in clinical practice. Multiple studies have underscored concerns about potential disruptions of clinical workflows, primarily due to time constraints and disagreements regarding the optimal content and format of DAs.^7^ Many of these challenges are linked to healthcare professionals, who play a critical role in the effective utilization of DAs, and to the organizational contexts in which they work. However, there is a notable scarcity of literature that explores their perspectives and perceptions regarding the utilization of DAs in the dynamic and intricate decision‐making process about forgoing cancer specific treatment in patients with advanced cancer.^8^

Therefore, the primary research questions of this study were:

1. What are the perceived challenges and barriers encountered by healthcare providers during the implementation of a DA for the care of patients with advanced cancer on decisions to forgo or continue cancer specific treatment in a outpatients' settings of a comprehensive cancer center?
2. What are the institutional factors and settings that contribute to the successful implementation of a DA for patients with advanced cancer on decisions to forgo cancer specific treatment in outpatient setting within a university hospital?

## 2 METHODS

### 2.1 Decision aid

Our goal was to develop a universally applicable, customizable DA for patients with various types of advanced cancer who are facing decisions whether to continue or forgo further anticancer treatment. Drawing on insights from qualitative interviews (first dataset), focus groups (second dataset), we designed and implemented a two‐part DA. Additionally, we integrated findings from a literature review and gathered patient perspectives through 16 qualitative face‐to‐face interviews focused on the DA's content.^9^ The DA was developed collaboratively with all project partners, including diverse disciplines such as medical ethics, palliative care, oncology, social sciences, and health services research, adhering to the criteria by the International Patient Decision Aid Standards (IPDAS).^10^

The DA consists of two parts which can be applied on different time points as disease progresses: the first part is provided to patients with incurable cancer when the first‐line therapy started. It contains general inquiries about information needs and is thought to support the formation patients' own opinion on life goals, values and preferences if faced with a dire prognosis. This part of the DA can be used either by patients alone at home to help them reflect on their values and preferences or during consultations between a patient and their treating oncologist.

The second part addresses the decision about continuing or forgoing anticancer therapy. It provides arguments both for and against each option and includes blank spaces for the oncologist to complete prognostic data such as median survival and the percentage of patients who may benefit from further treatment.

This second part is used in disease progression because it raises the crucial question of whether therapy should be continued or not–linking it to conversations about the results of staging examinations in advanced disease. Hence, the second part of the DA is completed during the staging interview with the physician and is intended to provide the patient with individualized information about their prognosis, possible treatment options and their chances including forgoing anticancer treatment and best supportive care. Hence, one element of the DA was that the prognosis and prospects of success of the different options must be clarified by the junior physician with the senior physician before the conversation with the patient. By tailoring the timing of DA administration based on disease‐specific considerations and disease progression, we aimed to ensure that patients could be step by step prepared for informed decisions about their care. The timeline of the use of the DA is presented in the Table 1.

**TABLE 1:** The Timeline for the use of the DA.

DA part I on patients’ values and preferences	Provided to patients with incurable cancer prior to their staging evaluation following the failure of first‐line therapy. It can be used independently by the patient
DA part II on treatment options	Provided to patients who are under the last line of guideline‐based therapy (depending on the cancer type) after their staging examination. The DA is used for the conversation on staging results and further strategy between the patient and their treating oncologist

### 2.2 Data collection

This study used qualitative data sets from two related projects, which both focused on the development and implementation of decision‐making tools for oncologists and patients with advanced cancer.

### 2.3 Dataset 1

The first sample comprised qualitative semi‐structured face‐to face‐interviews with oncologists from different units and specialties to explore the specific circumstances, barriers, and necessary support for the adaptation and implementation of a standard ethical guideline on therapy limitation near the end of life into clinical practice.^11^ The interviews were carried out from October 2018 to February 2019. On average, interviews lasted 35 min. The demographic data of the interviews' participants is presented in Table 2.

**TABLE 2:** Sample description.

Dataset 1	Mean (range)/*n*
Interviews (*n* = 24)	
Age	38 years (28–53 years)
Work experience	10 years (5 month–36 years)
Male	8
Female	16
Profession & position	Junior physician; senior physicians, section heads, clinic director (Fields: Medical oncology, dermato‐oncology, Gynecologic oncology, radiation oncology, Translational Oncology, Palliative Medicine)

PETUPAL project dataset 2	
Focus group 1 Heidelberg (*n* = 9)	
Age (mean)	45.1 years (27–61 years)
Work experience in oncology	17 years (14–20 years)
Male	2
Female	7
Profession	Social pedagogue; Social worker; Study coordinator, Sport trainer; Nurse, Pain Nurse; Senior Anaesthetist (Intensive/Palliative Care); Assistant physicians; Nurse
Focus group 2 Heidelberg (*n* = 4)	
Age (mean)	35.7 (32–43 years)
Work experience	8.25 years (6–13 years)
Male	1
Female	3
Profession	junior physicians
Focus group 3 Jena (*n* = 6)	
Age	37 years (30–55 years)
Work experience	7.1 years (5 months–24 years)
Male	4
Female	2
Profession	Junior physician; Senior physicians
DA‘ trial interviews (*n* = 5)	
Age	30 years (27–33 years)
Work experience	3.1 years (6 months–7 years)
Male	3
Female	2
Discussions with DA use (range)	4–10
Profession	Junior physicians

### 2.4 Dataset 2

The PETUPAL‐study (Preference‐based DA to support participatory decisions about tumor‐specific and palliative therapy in the last months of life), aimed to develop and implement a generic DA for advanced cancer patients, specifically addressing the question of continuing or forgoing tumor‐specific therapy.^9^ This study was conducted at two German centers: National Center for Tumor Diseases, University Hospital in Heidelberg, and the Department of Internal Medicine II (Palliative Care), University of Jena. The published study protocol can be accessed for further details of the study.^9^

Prior to the development of the DA, three semi‐structured face‐to‐face focus groups with health care providers (*n* = 16) were conducted in Heidelberg and Jena.^9^ The demographic data of the focus groups is presented in Table 2.

The focus groups aimed to explore the challenges they encountered when discussing tumor‐specific versus palliative therapy with patients, strategies for implementing the DA, potential barriers, and the optimal timing for utilizing the DA.^9^ Purposeful sampling strategy was applied to insure variability of sample. Participants were recruited per telephone or via personal contact by a study nurse. The focus groups lasted approximately 90 min (each focus group).^9^ The focus groups were audio recorded and were subsequently transcribed.

After the DA was developed and implemented, we conducted five semi‐structured face‐to‐face interviews with oncologists who had used the DA with their patients in a real‐world clinical setting. The interview guide for these post‐implementation interviews was specifically designed by the research team to explore oncologists' perceptions of the DA's usability, its impact on patient care, and any challenges encountered during its use. For the post‐implementation phase, we approached 16 oncologists who had collectively participated in 34 discussions using the DA. Out of these, five oncologists agreed to participate in the interviews.

Interviews were audio‐recorded, transcribed verbatim, and analyzed using content analysis to identify themes related to the DA's effectiveness, practical utility, and integration into clinical workflows.

We used both interviews and focus groups with overall *n* = 48 participants to leverage their distinct advantages for a comprehensive understanding of the DA development and implementation. Interviews offered in‐depth, individualized insights from oncologists, allowing us to explore personal experiences, nuanced perspectives, and specific challenges in using the DA. Focus Groups facilitated interactive discussions among healthcare professionals, generating rich, multi‐faceted data through group dynamics and collective deliberation, and opinion‐building. The qualitative studies adhered to the guidelines outlined in the Consolidated Criteria for Reporting Qualitative Research (COREQ) framework.^12^

### 2.5 Analysis of the data

For the analysis of interviews and focus groups, we employed qualitative inductive content analysis following Sandelowski's approach.^13^ Two research team members (KL and RB) coded the data. The initial phase of the inductive content analysis encompassed open coding, wherein sentences or paragraphs were attributed with significance. Following this, the interconnections among the open codes were meticulously scrutinized to formulate cohesive categories. To ensure the rigor of the categorization process, collaborative discussions were held with fellow researchers. With the categories solidified, the subsequent stage involved the transformation of these categories into overarching, more generalized themes. The ensuing generalized themes were subjected to thorough scrutiny and refinement through discussions with colleagues possessing diverse expertise across various fields such as psychology, medicine, and social sciences. The analytical category system for the content analysis, including main themes, subcategories, and codes, is presented in Table 3.

**TABLE 3:** Analytical category system of the content analysis including main categories, subcategories and codes.

Themes	Category	Code
Role of the DA in advanced cancer	Positive impact of DA on patients care	DA is a valuable tool for patients It shows treatment alternatives to patient Facilitation of EOL conversation Patient can go into the conversation prepared; Help when doctor changes It helps structuring of conversation for patients; Consistently distribution of information to each patient Help patients to initiate discussions about forgoing cancer specific treatment
	Positive impact of DA on oncologists and their conversation with patients	Easier for the doctor to conduct conversation It helps structuring of conversation for oncologists
	Possible negative impact	Can lead to distrust between oncologist and patient
Tension between standardizing the use of a DA and responding to Individual patient needs	Standardizing the Use of a DA	There should be a standard in using DA Important is an institutional embeddedness There should be specific criteria for the DA use There should be specific criteria for timing There should be the mandatory use of a DA by all oncologists
	Benefits of standardization	Consistently timely distribution of information to each patient
	Overcoming Individual and Institutional Barriers to Timely Decisions of forgoing cancer‐specific treatment	Overcoming reluctance of oncologists to start discussions of forgoing cancer‐specific treatment Avoiding of heterogenous handling of the DA
	Challenges within the hierarchical system of a university oncology setting	Hierarchical structure in German and huge role of senior physician in decision‐making
	Education and organization‐related factors	Patients are seeking second opinions ➔ high expectations and pressure placed on oncologists not to talk about forgoing cancer‐specific treatment Frequent rotation of physicians in the outpatient setting of a university hospital Divergent opinions among the oncologists' team Lack of knowledge about possibilities of palliative care Death is still a taboo subject
	Should be not mandatory and used individually	DA as a flexible approach DA implementation only as a moral duty Should not be an obligation ➔ Unacceptance by doctors You must always adapt to patient No fixed plan during the conversation ➔ flexibility
	Uncertainty about patients	Various conditions for patients Unique and complex situation of patients Different coping styles with the disease Patient's current psychological state Readiness for self‐reflection Patients who are “ready” Difference in patients personalities
	Uncertainty about medical conditions	Uncertainty about prognosis Uncertainty about the treatment options
	Uncertainty about timing	Continuity is important Address and evaluate again and again Not at the beginning but after a few options Not at the last conversation Not to wait till all the options are exhausted When some options have already been exhausted As early as possible Not in the beginning

Impact of wrong timing	Wrong timing of DA could demoralize patients Wrong timing of DA could destroy hope
Prerequisites for the use of the DA	Support of senior oncologists	EOL interview should be conducted by someone with experience Role of the senior physician: acceptance if only accepted at senior physician level Involving senior physicians would be good >the senior physicians should be involved at the beginning
	Training in palliative medicine and DA use	Physician should be "well prepared" in all DA topics Do not leave young colleagues alone with a conversation Experience is important
	Establishing Collaborative Networks with Palliative Colleagues	Exchange between colleagues is missing Palliative care stigmatized Training of younger colleagues Interdisciplinary networking: involving palliative medicine in time
	Creating Suitable Conditions for the DA Use	University: difficult setting Excessive work demands on junior doctors Creating the right framework for a conversation: establish dedicated time slots with no phone interruption Time shortage➔ increasing time for consultations with patients

Date Analysis was done with the help of the data analysis software MAXQDA (VERBI GmbH, Berlin, Germany).

## 3 RESULTS

We identified two overall themes that are relevant for integration of DAs in outpatient advanced cancer care: (1) the tension between standardizing the use of DA and the responding to individual patient needs; (2) crucial factors facilitating the use of the DA such as the role of senior physicians, importance of training, and creating suitable conditions.

### 3.1 Tension between standardizing the use of a DA and the responding to individual patient needs

Despite an underlying consensus regarding the valuable role of decisional tools for patients, opinions differed regarding the optimal utilization of the DA, particularly in the context of decisions about forgoing tumor‐specific therapy and the uncertainty surrounding the optimal timing of such discussions.

One of the most contentious aspects highlighted by participants was the extent of standardization of the DA use. In the following, we present and analyze the two contrasting positions, as well as our efforts to find a middle ground between the two approaches: standardized use of DA and DA use at the discretion of the oncologists.

### 3.2 Standardization as response to individual and institutional barriers to timely decisions for or against tumor‐specific treatment

A part of participants emphasized the significance of institutional embeddedness for the successful implementation of a DA in an outpatient clinic particularly in context of advanced cancer care. They proposed the development of standardized guidelines to ensure the use of a DA by defining specific criteria for its appropriate application and timing. Furthermore, it was suggested that the mandatory use of a DA by all oncologists would ensure that advanced care planning information is consistently offered to each patient.

The absence of a sufficient standardization carries the risk of a very heterogenous handling of the subject matter. Some prioritize honesty by addressing the limited options, while others are reluctant to address forgoing treatments, ultimately diverting attention from the crucial focus on palliative care:

*And it might be easier to establish it if you define it as a sort of rule*. *Otherwise*, *you are always (laughs) yes*, *otherwise there's the good doctor and the bad doctor*: *the bad one talks about treatment limitation*, *and the good one offers another study*. *If you make it binding for everyone*, *at least to some extent*, *as a guideline*, *then it makes sense*. (Interview 3)

The proponents of the approach argue that standardizing the use of DA would be beneficial in tackling persistent challenges related to the care for patients with advanced cancer. Among these challenges, a significant concern identified by most participants is the hesitation of oncologists to engage in end‐of‐life discussions and the lack of knowledge about possibilities of palliative care. Several interviewees noticed that some oncologists tend to offer therapies, reflecting their persistent focus on emerging targeted therapies and their deeply held belief that they must always offer some form of treatment to patients, even in cases where benefits are unlikely or very limited. This inclination is further reinforced by the high expectations and pressure placed on them, particularly by patients seeking second opinions:

*Patients who come to our center specifically often seem less willing to accept that I can no longer offer a meaningful oncological therapy*. *So*, *I do believe that it is more challenging here for us*. (Interview 14)

Education and organization‐related factors, including the frequent rotation of physicians in the outpatient setting of a university hospital, result in challenges such as disrupted patient care continuity. This disruption can lead to a reduced sense of responsibility for patients and an increased workload for junior physicians:

*I believe one of the problems is the constant change of doctors*. *When patients don't consistently talk to the same person*, *it's also not possible to build a relationship*, *and then no one really feels responsible for the patient*. *That's a problem*. (Interview 18)

Lastly, the existence of divergent opinions among the oncologists' team, combined with the hierarchical structure in German healthcare where junior doctors have little autonomy, frequently results in a lack of coherence when it comes to making treatment decisions regarding the discontinuation of cancer‐specific treatments. This situation can impede the effective utilization of DAs.

This hindrance becomes particularly apparent when senior physicians hold divergent views on treatment approaches:

*Yes*, *but the thing is*, *when you have discussed BSC (best supportive care) because it was also the patient's wish*, *it can happen that you then hear from above*, *I don't agree with that*. (Focus group III)

The standardization and institutional embeddedness of the use of DAs were seen as potential solutions to these complex challenges within the hierarchical system of a university oncology setting.

Additionally, the implementation of standardized use of DAs to facilitate end‐of‐life conversations can alleviate the burden placed on patients when it comes to initiating and accepting these discussions. As one participant aptly expressed:

*Of course*, *the idea of standardizing this*, *so to speak*, *the idea that it's naturally asked about in routine and has a less shocking dimension for the patient*, *would be helpful if you communicate*: *This is part of a comprehensive approach for all our patients*. (Interview_08).

### 3.3 DA use upon the discretion of oncologists

Some participants, however, were more reluctant to standardize the use of the DA, primarily due to the unique and complex situation of patients with advanced cancer: the uncertainty about prognosis, and the treatment options, patients' differences in coping styles with the disease, their reluctance regarding end‐of‐life discussions create a complex landscape where individualized and tailored approaches are often necessary. They saw many obstacles to standardized use of the DA. In their perspective, the utilization of DAs should not only be individualized, taking into account a patient's current psychological and mental state and their readiness for self‐reflection, but also the decision as to whether DA is offered at all should be left to the oncologists' discretion. Some participants proposed that DAs should be provided exclusively to patients who are “ready” to use them. Consequently, in this approach, oncologists would determine whom to offer the DA to:

*I always find it difficult when patients have unrealistic treatment goals*. *We try to inform them about the status of their illness*, *but they insist on wanting extensive treatment and more time*. *They don't let realistic information sink in*. *I find it much harder to make decisions in such cases compared to when we can openly discuss these matters*. (Focus Group II)

The arguments for and contra standardizing the use of DA in oncology are presented in the Table 4.

**TABLE 4:** Arguments for and contra standardizing the use of DA in oncology near the end of life.

Arguments	Description of arguments	How we address the arguments
Arguments for standardizing the use of a DA for FAC patients		
Consistency	Patients' engagement in reflection regarding their preferences, values, and goals of care	Part 1 of the DA should be admitted to all patients who meet the specified inclusion criteria
Overcoming reluctance	Effectively tackling the prevalent reluctance among oncologists to engage in discussions about forgoing cancer‐specific treatments	
Enhanced patients' preparedness for discussions	Effective preparation for discussions with their oncologist and the subsequent decision‐making process	
Arguments against standardizing the use of a DA		
Patient variability	Unique patient situations and preferences in advanced cancer Some feel overwhelmed, especially with unrealistic goals	The administration of Part 2 of the DA can be customized to meet the specific situation and needs of each patient
Timing consideration	Challenging to determine optimal DA timing Patients vary in coping and self‐reflection readiness	
Organizational challenges	Lack of patient care continuity due to constant physician rotation	

Furthermore, within this approach there was no clear view on the timing of DA administration to patients. This uncertainty about appropriate timing was explained by difference in patients' personalities and coping strategies. Oncologists were concerned that wrong timing of DA could demoralize patients and would destroy hope. Opinions regarding the right timing for offering the DA ranged from “as early as possible” to “over and over again and not only once” to “not in the beginning,” “when some options have already been exhausted”:

Member 1 of the focus group: Well, I don't think that someone who receives an initial diagnosis that is already palliative should be given a DA. I believe that would be inappropriate. Rather, it should be given when several options have already been explored […]. (Focus group HD)

### 3.4 Prerequisites for the use of the DA

Despite differing viewpoints on the extent of standardization of the DA, all participants agreed on the existence of key factors that are essential for the successful implementation and utilization of the DA in oncology care.

#### 3.4.1 Support of senior oncologists

Most participants recognized that the successful implementation of the DA hinges on the support and willingness of senior physicians at the executive level. Senior physicians were seen as pivotal figures, advocating for and supporting junior oncologists in this process: “Senior physicians have to be willing to do it.” ; “You need role models.” (Int.6)

Furthermore, the experience of senior or more advanced doctors is a valuable resource for junior doctors. Many respondents described learning from them as particularly important:

*I learned those conversations because someone went in there with me*. *An experienced junior doctor or a senior doctor*. *They conducted these conversations themselves and I had the chance to learn from the model what is important*: *the choice of words and the whole attitude*. (Interview_09)

#### 3.4.2 The importance of training in palliative medicine and establishing collaborative networks with palliative colleagues

One of the crucial factors for successful DA implementation lies in regular training to acquire knowledge and experience in end‐of‐life planning, palliative medicine, supportive care, communication skills, and the utilization of DAs within the context of advanced cancer. Education on supportive therapy, coupled with establishing a network of colleagues in palliative medicine, was recognized as highly beneficial:

…*before we even think about handing such a form to a patient*, *we all need to extensively practice using it*, *I mean every one of us*. (Focus Group III)

#### 3.4.3 Creating suitable conditions for the DA use

Most participants highlighted the urgent need to establish suitable conditions for the effective utilization of DA with patients—meaning to establish dedicated time slots for these discussions, ensuring that oncologists are not interrupted or distracted:

*Framework conditions would include having undisturbed conversations*, *without the phone constantly ringing or someone constantly entering the room and demanding something*. (Interview 2)

## 4 DISCUSSION

Our results revealed a tension between the need for standardization as well as the need for flexibility in terms of content, timing, and the provision of the DA to patients. In order to respond and partly resolve this tension, we divided the DA in two parts for two different time points and introduced a customizable part on the clinical benefits and burdens of the next cancer‐specific line of therapy versus forgoing anticancer treatment and focusing on best supportive care.

By implementing this two‐part DA approach, our goal was twofold. Firstly, with the first part we aimed to gradually involve patients in a reflective process, allowing them to carefully consider their preferences and values in relation to their further treatment options, facilitating a more informed decision‐making process.

Secondly, we intended to combine the advantages of standardization through part one and still leave enough freedom to adapt the second part to the individual information needs of the patient. This standardized approach with a certain degree of flexibility for oncologists and patients aimed to support feasibility and acceptability of the DA. Furthermore, it allows more patient‐tailored approach, facilitating a step‐by‐step patient preparation process for decision‐making. Furthermore, it offers flexibility to tailor the DA to each patient's unique situation, prognosis, and preferences, reaching even those who initially avoid a realistic understanding of their disease by introducing information gradually.

To our knowledge, this represents the first customizable DA designed to offer flexibility across all types of advanced cancer informing the decision about continuing or forgoing cancer specific therapy.

Participants proposed standardization as a potential solution to address several identified challenges. One such challenge is therapeutic bias, where oncologists may prefer offering new treatment options and enrolling patients in clinical trials. since this does not require to necessarily address poor prognosis. Lack of knowledge about palliative care among oncologists, lack of continuity of patient care and hierarchical organizational structures were also arguments in favor for standardization.

Certain barriers have already been acknowledged and discussed in the existing body of literature concerning the implementation of DAs in cancer care.^14^ For example, a qualitative investigation involving advanced lung cancer patients and oncologists underscored that maintaining continuity of patient care forms a fundamental cornerstone for effective decision‐making.^15^ Nonetheless, achieving this continuity often proves to be challenging in outpatient settings within a university hospital environment. To tackle this issue, a potential solution that emerged in our study was the standardized utilization of DAs. This approach would enable transitioning treating physicians to access the completed DAs, thus addressing the continuity challenge.^16^

Furthermore, it is crucial to acknowledge that far advanced cancer and handling a limited prognosis is a unique and distinct context. As outlined by other participants, a standardized implementation of DA may not be ideally suited for this setting. Instead, the DA was perceived as a conceptual approach, advocating for adaptability to align with each patient's specific circumstances, emotional state, and readiness for self‐reflection. This perspective highlights the importance of tailoring DA usage to meet the individual needs and preferences of patients within the context of far advanced cancer.

Also, existing literature highlights the complexities of the internal factors within the life‐threatening context when it comes to incorporating DAs into regular clinical practice. In these situations, oncologists often prioritize addressing patients' immediate care and emotional needs, which can overshadow the importance of providing decision support.^17^ Combining the desired effects of standardization with the need for flexible solutions, we introduced a two‐part customizable DA that can be used for all cancer entities and allow due to its design as a two‐part document certain flexibility and consideration of patients' individuality.

Furthermore, three essential factors for feasibility and acceptability of the DA were identified in our study: Support of senior oncologists; creating suitable conditions for the DA use and the importance of training in palliative medicine and establishing collaborative networks with palliative colleagues.

The support of senior oncologists plays a pivotal role within our context, a phenomenon inherently tied to the stringent hierarchical structure characteristic of German medical system. This fact underscores a critical aspect with implications both advantageous and challenging in the realm of innovation implementation.

On the one hand, the hierarchical structure of German hospital settings can provide a framework for efficient decision‐making, enabling a top‐down approach for introducing innovations such as the DA. However, it also means that securing the endorsement and active engagement of senior oncologists is paramount for the successful integration of the DA within the context of German hospitals.

Since we learned that the support and involvement of senior physicians plays a crucial role in ensuring consistent implementation of DAs within the oncology team, right from the outset of the study, senior oncologists were actively engaged in the development process of the DA, thereby enhancing their willingness to promote its utilization among junior doctors.

Yet, even in their absence, additional measures are required to effectively implement DAs in practice. Among these strategies, comprehensive training in DA use and practical support stands out—underscored also by diverse research on DA implementation.^14^

Moreover, fostering connections with peers in the realm of palliative medicine and ensuring thorough documentation of the conducted discussions emerge as significant contributors to the successful introduction and sustained usage of DAs.

### 4.1 Study strengths and limitations

One of the strengths of this qualitative study is its large sample size. By combining two complementary datasets, we achieved a total of *n* = 19 participants from three focus groups and *n* = 29 interview participants (*n* = 24 interviews from dataset 1 and *n* = 5 interviews with oncologists who used the DA (dataset 2)).

A further strength of this study lies in its adept integration of diverse perspectives. The conducted interviews and focus groups effectively capture insights from a broad spectrum of healthcare professionals, encompassing physicians, social services professionals, palliative care specialists, and nurses. This holistic approach contributes to a nuanced comprehension of the inherent challenges associated with the implementation of DA in the context of far advanced cancer.

The utilization of the DA occurred within the context of a study trial phase, where participating oncologists were provided with instructions on how to effectively employ the DA during their patient discussions. Although the study aimed to simulate a setting closely resembling real‐life scenarios, it is worth noting that all participants could have potentially been influenced by their involvement in this trial and specific instructions on how to use the DA, introducing a certain degree of bias.

## 5 CONCLUSION

The debate concerning the right balance between standardized utilization and individually tailored application of DA has surfaced as a particularly contentious focal point in our qualitative analysis. In response, we developed an innovative two‐part customizable DA for patients with different cancer types. While the first part can be offered on a standardized base, the use of the second part can be customized based on each patient's unique circumstances, course of disease and preferences.

## AUTHOR CONTRIBUTIONS

**K. Laryionava:** Conceptualization (equal); formal analysis (equal); investigation (equal); methodology (equal); project administration (lead); software (equal); writing – original draft (equal); writing – review and editing (equal). **R. Boekels:** Formal analysis (equal); methodology (equal); validation (supporting); writing – original draft (supporting). **J. Schildmann:** Conceptualization (supporting); formal analysis (supporting); funding acquisition (supporting); investigation (supporting); methodology (supporting); project administration (supporting); supervision (equal); writing – review and editing (supporting). **M. Wensing:** Conceptualization (equal); formal analysis (equal); methodology (supporting); project administration (supporting); validation (equal); writing – review and editing (equal). **U. Wedding:** Conceptualization (equal); funding acquisition (lead); investigation (equal); methodology (supporting); project administration (supporting); writing – original draft (supporting); writing – review and editing (equal). **B. Surmann:** Conceptualization (equal); data curation (supporting); methodology (equal). **K. Mehlis:** Methodology (equal); validation (equal). **C. Gebel:** Methodology (equal); validation (equal); writing – review and editing (equal). **M. Cinci:** Data curation (equal); formal analysis (equal); resources (equal); supervision (supporting); writing – review and editing (supporting). **K. Krug:** Methodology (equal); validation (equal); writing – review and editing (equal). **E. C. Winkler:** Conceptualization (equal); funding acquisition (lead); methodology (equal); project administration (lead); writing – original draft (equal); writing – review and editing (equal).

## FUNDING INFORMATION

This study is supported by research grant Nr. 70,113,243 of the Deutsche Krebshilfe (German Cancer Aid).

## CONFLICT OF INTEREST STATEMENT

The authors declare that they have no competing interests.

## ETHICS STATEMENT

This study was approved by the Ethics Committee of the Medical Faculty of the University of Heidelberg and University of Jena. Written informed consent was obtained from all participants of the study.

## TRIAL REGISTRATION

ClinicalTrials.gov NCT04606238; https://clinicaltrials.gov/ct2/show/NCT04606238.

## Data Availability

The data that support the findings of this study are available on request from the corresponding author (ECWinkler). The data are not publicly available due to privacy or ethical restrictions.
